# Massachusetts General Hospital

**Published:** 1850-04

**Authors:** 


*Massachusetts General Hospital.*—Whosoever has examined, in detail, this magnificent establishment must admit that it has no rival in America; and where is its superior in Europe? In 1849, there were admitted 870 patients; viz: 531 males and 339 females; 84 died, 432 recovered; 126 were much releived. The greatest number at any one time, was 127; greatest number of free patients, 89; average number of patients, 112; average number of paying patients, 26; whole number of paying patents, 273. The whole number of admissions exceed those of the previous year, by 66. Expenses, $29,391 30; of which $4,788 12 was paid by patients. The gross amount of the annual expenses, divided by the average number of patients, will give $4 55 as the weekly expense of each patient.—*Boston Med. anr Surg. Journal.*

